# Long-term impact of SARS-CoV-2 infection on cardiorespiratory fitness: a meta-analysis

**DOI:** 10.3389/fpubh.2023.1215486

**Published:** 2023-10-18

**Authors:** Busaba Chuatrakoon, Supatcha Konghakote, Piangkwan Sa-nguanmoo, Sothida Nantakool

**Affiliations:** ^1^Department of Physical Therapy, Faculty of Associated Medical Sciences, Chiang Mai University, Chiang Mai, Thailand; ^2^Environmental-Occupational Health Sciences and Non Communicable Diseases Research Center, Research Institute for Health Sciences, Chiang Mai University, Chiang Mai, Thailand

**Keywords:** age, COVID-19, cardiorespiratory fitness, meta-analysis, peak oxygen uptake, SAR-CoV-2, symptom

## Abstract

**Background:**

Despite surviving Coronavirus disease 2019 (COVID-19), its long-term impact is of concern. Low cardiorespiratory fitness is a strong predictor of all-cause mortality, and likely affected by multisystem impairments following COVID-19 infection. Accumulating evidence has identified the impact of COVID-19 on cardiorespiratory fitness level. However, the findings have been controversial. Conclusive evidence is still needed.

**Objectives:**

This review aimed to systematically summarize and synthesize whether the SARS-CoV-2 infection diminishes cardiorespiratory fitness in COVID-19 survivors.

**Design:**

The study design was a systematic review and meta-analysis.

**Methods:**

A search was carried out using PubMed, CINAHL, Scopus, Embase and the Cochrane Library, together with reference lists (searching from their inception to January 2023). Observational studies investigating the impact of COVID-19 on outcomes relevant to cardiorespiratory fitness (i.e., peak oxygen uptake) were included. Weighted mean difference (WMD) and 95% confidence interval (CI) were used to identify a pooled effect estimate. Use of a random effects model was considered as the main method. Grading of Recommendation Assessment, Development and Evaluation approach was employed to determine the certainty of evidence. This meta-analysis was registered with PROSPERO (registration number: CRD42023393108).

**Results:**

Seven eligible studies (4 cross-sectional, 2 cohort, and 1 case–control studies) involving 4,773 participants were included in this meta-analysis. A pooled effect estimates showed that patients in the surviving COVID-19 group had a significant reduction in peak oxygen uptake when compared to their counterparts in the non-COVID-19 group (WMD −6.70, 95%CI −9.34 to −4.06, low certainty). A subgroup analysis by age found that COVID-19 survivors in the young- to middle-aged and middle- to older-aged subgroups had significant reductions in peak oxygen uptake when compared to their counterparts in the non-COVID-19 group (WMD −5.31, 95%CI −7.69 to −2.94, low certainty; WMD −15.63, 95%CI −28.50 to −2.75, very low certainty, respectively). Subgroup analyses by symptom found that patients with moderate to severe symptoms in the surviving COVID-19 group had significantly lower peak oxygen uptake than their counterparts in the non-COVID-19 group (WMD −15.63, 95%CI −28.50 to −2.75, very low certainty).

**Conclusion:**

The current meta-analysis concluded that patients in the COVID-19 survivors had poorer cardiorespiratory fitness than their counterparts in the non-COVID-19 group, but there is considerable uncertainty of evidence. Poorer cardiorespiratory fitness is likely to be more pronounced in COVID-19 survivors who are getting older and had severe symptoms, but it is uncertain whether such finding has a valuable in clinical context.

**Systematic Review Registration:**

https://www.crd.york.ac.uk/PROSPERO/, CRD42023393108.

## 1. Introduction

Coronavirus disease 2019 (COVID-19), caused by the severe acute respiratory syndrome coronavirus 2 (SARS-CoV-2), has been of crucial health concern worldwide (1). A reported 757 million infected people, and more than 6 million deaths have been addressed from this pandemic (2). People infected with SARS-CoV-2 experience a wide range of symptoms, from asymptomatic to symptomatic (3). Since SARS-CoV-2 is the main pathogen of the human respiratory system, the most common clinical presentations include cough, fatigue and dyspnea (4). Apart from respiratory tract infection, COVID-19 plays a role in multiorgan impairment, such as impaired cardiac, vascular and skeletal muscle systems (5–7). Despite surviving COVID-19, its long-term impact is also of concern. Persistent symptoms that lasted for 3 months after the onset of illness are defined as long COVID-19 (8). Various reports have documented that long-term organ damages are attributed to immune-mediated response, inflammation, and viral persistence following COVID-19 infection (9). Such physiological alterations drive several specific long-term symptoms such as difficulty breathing, chest pain, tachycardia, and muscle weakness (10). Evidence has also shown a declined lung function in patients who recovered from COVID-19 infection, even in mild symptomatic and asymptomatic cases (11–13). In addition, a reduction in muscle strength has been observed in patients with long-COVID-19 syndrome (14). Approximately 36% of COVID-19 survivors experience long-term symptoms that persist at least 30 days after the onset of the COVID-19 infection (10) and some recovered patients undergo COVID-19-acquired complications (15). Persistence in these impairments is consequently linked to a reduction in functional capacity and decrease in quality of life (16, 17).

Cardiorespiratory fitness (CRF), used as a quantified measure of functional capacity, indicates an integrated function of respiratory, cardiovascular and musculoskeletal systems. It is well-established that low CRF is a strong predictor of all-cause mortality, including cardiovascular disease (CVD) (18, 19). American Heart Association has suggested CRF measurement in clinical practice since it is not only an independently CVD risk predictor, but also a complemented factor to traditional risk to improve CVD risk prediction (19). According to this, CRF in long COVID-19 patients has been recognized increasingly. Due to the sequelae of long-term multisystem impairments following SARS-CoV-2 infection, patients with long COVID-19 are likely to undergo increased CVD risk as a consequence of reduced CRF. A recent evidence synthesis has documented that severe symptoms were associated with advancing age (20). In addition, a very recent study has revealed that the more worsening symptoms, the lower CRF level (21). As the evidence suggests, it is likely that older age is a driving factor in further reducing CRF. A growing body of evidence has identified the impact of COVID-19 on CRF level. However, findings among existing studies have been controversial. While some evidence has revealed a decrease in CRF in confirmed COVID-19 patients (22, 23), other proof has failed to confirm low CRF in this group of patients (24). Conclusive evidence as to whether COVID-19 compromises the level of CRF is still needed. Thus, this study mainly aimed to systematically summarize and synthesize whether the SARS-CoV-2 infection diminishes CRF in COVID-19 survivors. Based on the evidence, we hypothesized that COVID-19 survivors would have a lower CRF level than individuals with non-COVID-19, especially in older age and severe symptom subgroups.

## 2. Methods

### 2.1. Search strategy

The systematic review and meta-analysis proceeded in accordance with the Preferred Reporting Items for Systematic reviews and Meta-Analyses (PRISMA) guidelines (Supplementary Table S1) (25). Five search engines including PubMed, CINAHL, Scopus, Embase, and the Cochrane Library (from their inception to January 2023), together with reference lists were used. All relevant studies were identified using the following search terms: (“covid” OR “coronavirus”) AND (“cardiorespiratory fitness” OR “physical fitness” OR “cardiopulmonary fitness” OR “fitness performance” OR “cardiorespiratory performance”) (Supplementary Table S2). This meta-analysis was registered in the PROSPERO database (https://www.crd.york.ac.uk/PROSPERO/, registration number: CRD42023393108).

### 2.2. Study selection

BC and SK screened the title and abstract independently. To obtain eligible studies, all of them were screened based on the PICOS criteria: We included observational studies investigating the impact of COVID-19 on outcomes relevant to CRF [i.e., peak oxygen uptake (VO_2peak_)], participants aged >18 years old, no gender restrictions, no language restrictions, and both published and unpublished studies. Studies conducted in an animal model and other study designs were excluded (Supplementary Table S3). Any disagreement between two reviewers was resolved by the third reviewer (SN).

### 2.3. Risk of bias (quality) assessment

Assessing risk of bias (ROB) was performed by BC and SK independently. The ROB of case–control and cohort studies were graded using the Newcastle-Ottawa Scale (NOS), while cross-sectional studies were evaluated using the Appraisal (AXIS) tool (26). The NOS contains eight items that are interpreted as good, fair or poor quality according to the scores assessed. The AXIS tool consists of 20-point questionnaires including five sections (introduction, methods, results, discussion and others) scored as “yes”, “no” or “do not know”. Discrepancy between the reviewers was resolved by the third reviewer (SN).

### 2.4. Assessment of certainty of evidence

Certainty of evidence was evaluated by two independent reviewers (BC and SK) using the Grading of Recommendation Assessment, Development and Evaluation (GRADE) approach (27). GRADE was determined based on the following domains: risk of bias, inconsistency, indirectness, imprecision, and publication bias. The certainty of evidence can be classified into four levels: high, moderate, low, and very low to indicate how confident that an effect estimate is close to the true effect.

### 2.5. Data extraction

SN and PS extracted the following information independently: author, year of publication, study design, participant details, continent, setting and CRF outcome (VO_2peak_). All extracted data were recorded in a Microsoft Excel program. Incomplete outcome data were resolved by sending an email to the authors of the study. If no response within a week, the incomplete publication was excluded. Any disagreement was resolved by consensus between the two reviewers.

### 2.6. Statistical analyses

CRF outcome was analyzed using a participant number, and mean and standard deviation (SD) in the exposure (i.e., confirmed COVID-19 group, outcome value after COVID-19 infection) and non-exposure groups (i.e., matched-control group, outcome value prior to COVID-19 infection). A pooled meta-analysis was identified by weighted mean difference (WMD) and 95% confidence interval (CI). Use of the random effect model was considered as a main method for analysis if there was substantial heterogeneity across studies presenting as *I^2^* > 50%, with a *p* value of Cochrane’s Q < 0.1 (28). Finding out possible sources of substantial heterogeneity was attempted by performing subgroup analysis, which was carried out by age ranges and symptoms. To ensure robustness, generating sensitivity analysis was considered based on unclear characteristics of the outcome (i.e., estimated value of VO_2peak_) and participants (i.e., unmatched-control participants). Publication bias was measured by funnel plot, contour-enhanced funnel plot, Begg’s test, and Egger’s test. All analyses were performed using STATA Statistical Software, version 14.2 (StataCorp LP, United States).

## 3. Results

**Figure 1:**
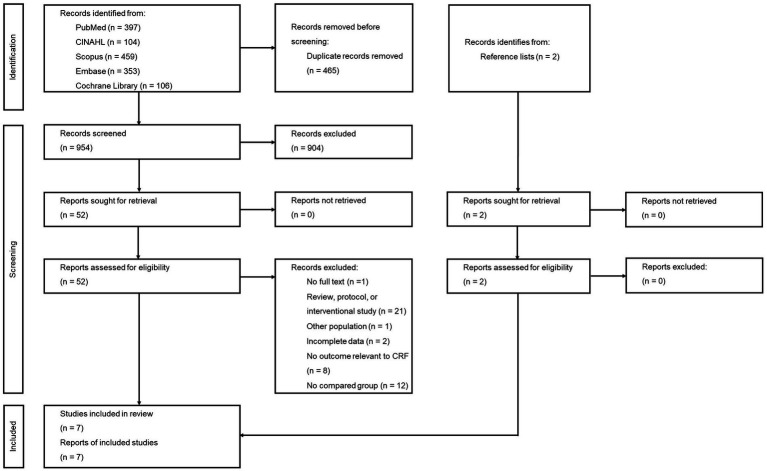
PRISMA flow diagram.

A total of 1,419 studies were retrieved from the five engine searches. Of these, 465 duplicates were removed and the 954 remaining studies were screened from title and abstract. Fifty remaining studies and two additional ones from reference lists were reviewed for more details. Among these, 45 were excluded due to no full text available (*n* = 1), other study types (*n* = 21), non-COVID-19 participants (*n* = 1), incomplete data (*n* = 2), no relevant outcome (*n* = 8), and no group for comparison (*n* = 12) (Supplementary Table S4). Thus, seven studies were finally eligible for meta-analysis. The PRISMA flow diagram is shown in Figure 1.

Details of the included studies are illustrated in Table 1. Seven eligible studies, involving 4,773 participants, were mostly cross-sectional (22, 24, 29, 30), while others were case–control (23), retrospective cohort (31, 32), and prospective cohort studies. The mean age of all participants was 40 years, and the majority were male (80.1%). Most of the studies were conducted in Europe (23, 24, 29–32), and one only in South America (22). The study settings comprised four studies containing hospital-based participants (22, 23, 29, 30), two including community-based participants (24, 31), and one that collected combined hospital and community-based participants (32). The symptoms of the participants varied, ranging from asymptomatic to severe symptomatic from COVID-19. All of the studies reported VO_2peak_ as CRF outcome (22, 24, 29, 30, 32), except for two that reported estimated VO_2peak_ (23, 31).

**Table 1:** Characteristics of eligible studies.

Author	Year	Study design	Characteristics of participants	Age range	Continent	Setting	COVID-19 symptoms and duration					Outcome and result
							Mean duration of hospitalization (days)	Mean duration of infection (days)	Symptoms during infection	Symptom duration after recovered- COVID (days)	Days from COVID to measures	
Back	2022	Cross-sectional	Sample size (CG/NCG): 29/18 Male (CG/NCG): 15/9 Age (CG/NCG) (yr, mean ± sd): 40 ± 11/38 ± 13	Young and middle-aged	South America	Hospital	6	n/a	Mild to moderate	20	30	↓ VO_2peak_ in CG
Crameri	2020	Cross-sectional	Sample size (CG/NCG): 145/54 Male (CG/NCG): 126/48 Age (CG/NCG) (yr, mean ± sd): 20.8 ± 1.5	Young-aged	Europe	Community	n/a	n/a	Sym and asym	n/a	45	↔ VO_2peak_ in CG
Ekblom-Bak	2021	Case–control	Sample size (CG/NCG): 857/3426 Male (*n*) (CG/NCG): 603/2412 Age (yr, mean ± sd) (CG/NCG): 49.9 ± 10.7	Middle-aged	Europe	Hospital	n/a	n/a	Severe	n/a	n/a	↓ EstVO_2peak_ in CG
Gattoni	2022	Retrospective cohort	Sample size (CG): 13 Male (CG) (*n*): 13 Age (CG) (yr, mean ± sd): 23.9 ± 4.0	Young -aged	Europe	Community	n/a	15	Mild	100	n/a	↓ EstVO_2peak_ in CG
Ladlow	2022	Prospective cohort	Sample size (CG/NCG): 87/26 Male (CG/NCG): 74/22 Age (CG/NCG) (yr, mean ± sd): 39.6 ± 4.7	Young-aged	Europe	Hospital plus community	n/a	n/a	Mild to severe	n/a	159	↓ VO_2peak_ in CG
Pleguezuelos	2021	Cross-sectional	Sample size (CG/NCG): 15/15 Male (CG/NCG): 15/15 Age (CG/NCG) (yr, mean ± sd): 54.6 ± 9.1/52.2 ± 4.9	Middle-aged	Europe	Hospital	23.2	n/a	Severe	n/a	79	↓ VO_2peak_ in CG
Raman	2021	Cross-sectional	Sample size (CG/NCG): 58/30 Male (CG/NCG): 34/18 Age (CG/NCG) (yr, mean ± sd): 55.4 ± 13.2/53.9 ± 12.3	Middle and older-aged	Europe	Hospital	10.2	n/a	Moderate to severe	n/a	159	↓ VO_2peak_ in CG

asym, asymptomatic; CG, COVID-19 infected group; NCG, non-COVID-19 infected group; EstVO_2peak_, estimated VO_2peak_; Sym, symptomatic; ↓, significantly lower than NCG; ↔, not different from NCG.

### 3.1. Risk of bias (quality) assessment

Of four cross-sectional studies, one met 16 out of 20 assessment criteria (24), two met 15 (22, 30), and one met 14 (29) (Table 2). Of these, no study had provided sample size justification. For case–control and cohort studies, one had good quality (23), while the other two had fair quality (31, 32) (Table 3).

**Table 2:** Risk of bias (quality) assessment: appraisal tool for cross-sectional studies (AXIS).

Author	Study design	Introduction	Methods										Results					Discussion		Other	
		Objectives of the study clear	Study design appropriate	Sample size justified	Population clearly defined	Sample frame taken from an appropriate population	Selection process likely to appropriate participants	Address non-responders	Appropriate outcome	Outcome variables were measured appropriately	Clearly determined statistical significance	Methods sufficiently described	Basic data adequately described	Non-responders bias was concerned	Non-responders were described	Results internally consistent	Results presented all the analyses	Discussions and conclusions justified by the result	Limitations were mentioned	There were conflicts of interest	Ethical approval
Back	Cross-sectional	y	y	n	y	y	y	?	y	y	y	y	y	?	?	y	y	y	y	n	y
Crameri		y	y	n	y	y	y	y	y	y	y	n	y	y	y	y	y	y	n	n	y
Pleguezuelos		y	y	n	y	y	y	?	y	y	y	y	y	?	?	y	y	y	n	n	y
Raman		y	y	n	y	y	y	?	y	y	y	y	y	?	?	y	y	y	y	n	y

y = yes, n = no,? = Don’t know.

**Table 3:** Risk of bias (quality) assessment: the Newcastle-Ottawa Scale (NOS).

NOS for cohort study										
Author	Study design	Selection				Comparability	Outcome			Interpretation
		Representativeness of the exposed cohort	Selection of the non-exposed cohort	Ascertainment of exposure	Outcome was not present at start of study	Comparability of cohorts	Assessment	Follow-up long enough	Adequacy of follow up of cohorts	
Gattoni	Retrospective cohort	3	1	1	2	1	2	1	1	Fair
Ladlow	Prospective cohort	1	2	1	2	1	1	1	1	Fair

NOS for case–control study										
Author	Study design	Selection				Comparability	Exposure			Interpretation
		case definition adequately	Representativeness of the cases	Selection of Controls	Definition of Controls	Comparability of cases and controls	Ascertainment	Same method of ascertainment	Non-Response rate	
Ekblom-Bak	Case–control	2	1	1	1	1	1	1	2	Good

### 3.2. Long-term impact of COVID-19 on cardiorespiratory fitness

**Figure 2:**
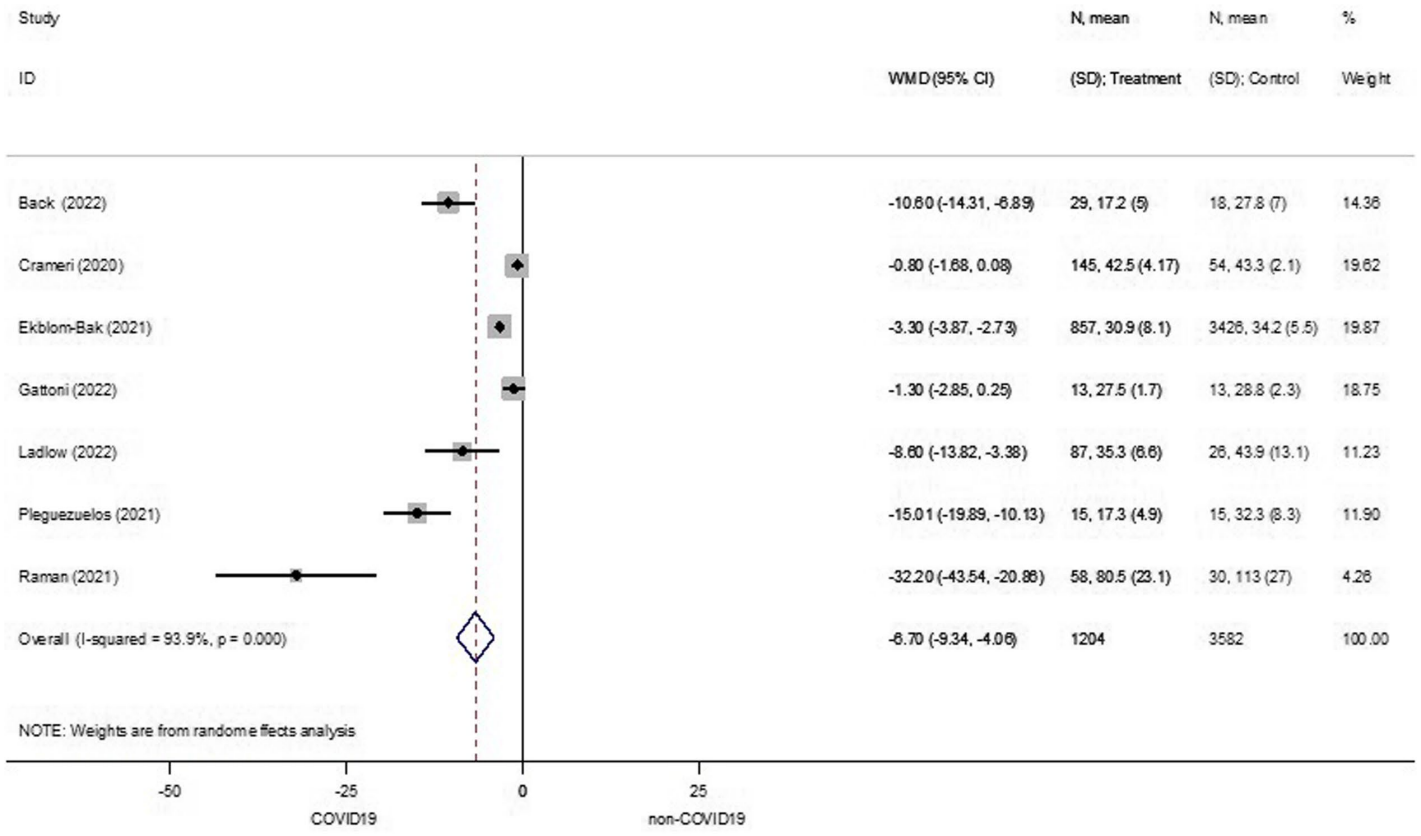
Cardiorespiratory fitness between COVID-19 and non-COVID-19 groups. ID, identification; *N*, number of participants; SD, standard deviation; WMD, weighted mean difference.

CRF was reported in all of the studies as amount of VO_2peak_. From the seven studies, pooled effect estimates showed that patients surviving COVID-19 (COVID-19 group) had a significant 6.70 milliliter (mL) reduction in VO_2peak_, compared to their counterparts in the non-COVID-19 group (WMD −6.70, 95%CI −9.34 to −4.06, *I^2^* 93.9%, 7 studies, low certainty) (Figure 2).

According to substantial heterogeneity across the studies, subgroup analyses were carried out based on age ranges and symptoms. Age ranges were separated into four subgroups, including young-aged, middle-aged, young to middle-aged, and middle to older aged. The young-aged subgroup in the COVID-19 group had a similar VO_2peak_ to their counterparts in the non-COVID-19 group (WMD −2.01, 95%CI −4.19 to 0.17, *I^2^* 76.3%, 3 studies, low certainty) (Supplementary Figure S1). In line with the young-aged subgroup, the middle-aged subgroup in the COVID-19 group had the same VO_2peak_ as their counterparts in the non-COVID-19 group (WMD −8.89, 95%CI −20.36 to 2.57, *I^2^* 95.4%, 2 studies, low certainty) (Supplementary Figure S1). The young- to middle-aged subgroup had a significant reduction of 5.31 mL in VO_2peak_ in the COVID-19 group when compared to the non-COVID-19 group (WMD −5.31, 95%CI −7.69 to −2.94, *I^2^* 93.1%, 6 studies, low certainty) (Supplementary Figure S2). The middle- to older-aged subgroup also had a significantly lower VO_2peak_ in the COVID-19 group when compared to the non-COVID-19 group (WMD −15.63, 95%CI −28.50 to −2.75, *I^2^* 95.7%, 3 studies, very low certainty) (Supplementary Figure S3). Subgroup analyses by symptom included mild to moderate, moderate to severe, and severe subgroups. Among the mild to moderate and severe subgroups, VO_2peak_ was equal between those in the COVID-19 and non-COVID-19 groups (WMD −5.79, 95%CI −14.90 to 3.32, *I^2^* 95.1%, 2 studies, low certainty; WMD −8.89, 95%CI −20.36 to 2.57, *I^2^* 95.4%, 2 studies, very low certainty, respectively) (Supplementary Figures S4, S5). However, patients who had moderate to severe COVID-19 symptoms had significantly lower VO_2peak_ than those in the non-COVID-19 group (WMD −15.63, 95%CI −28.50 to −2.75, *I^2^* 95.7%, 3 studies, very low certainty) (Supplementary Figure S6).

Two of the studies reported an estimated VO_2peak_ rather than a measured one. Due to uncertain characteristics of outcome, a sensitivity analysis was performed to ensure robustness by removing (23, 31). After these omissions, patients in the COVID-19 group had significantly reduced VO_2peak_ than their counterparts in the non-COVID-19 one (WMD −12.25, 95%CI −20.24 to −4.25, *I^2^* 95.5%, 5 studies, low certainty) (Supplementary Figure S7). Such sensitivity analysis showed a trend toward decreased VO_2peak_ in the COVID-19 group when compared to the non-COVID-19 one. Sensitivity analysis was performed further by eliminating (32), due to unmatched-control participants. After this exclusion, the pooled effect estimates revealed a significantly lower VO_2peak_ in the COVID-19 group than in the non-COVID one (WMD −6.43, 95%CI −9.21 to −3.66, *I^2^* 94.6%, 6 studies, low certainty) (Supplementary Figure S8).

### 3.3. Publication bias of the included studies

Assessment of publication bias of the studies was carried out (Supplementary Figure S9). There was a possible publication bias where asymmetry of funnel plot was expressed (Supplementary Figure S9A). However, contour-enhanced funnel plot revealed no publication bias, as most missing studies were in an area of high statistical significance (*p* < 0.01) (33) (Supplementary Figure S9B). In addition, Begg’s test and Egger’s test had no significant differences (*p* = 0.051 and 0.096, respectively) (Supplementary Figure S9C).

## 4. Discussion

This review aimed to systematically summarize and synthesize whether the SARS-CoV-2 infection diminishes CRF in patients who have recovered from COVID-19. The main findings of this review were (1) the COVID-19 survivors had poorer CRF when compared to the non-COVID-19 one, (2) poorer CRF was presented in the young- to middle-aged subgroup and was more pronounced in the middle- to older-aged subgroup, and (3) the COVID-19 survivors that had moderate to severe symptoms had lower CRF than the non-COVID-19 group, while the COVID-19 survivors with severe symptoms alone had a similar CRF level when compared with the non-COVID-19 group.

The long-term impact of COVID-19 on health status is pronounced despite recovering from it. Persistent multiorgan impairment is possibly associated with a declined CRF, which is a factor determining reduced functional ability, poor quality of life (16, 17), and increased all-cause mortality (18, 19). The meta-analysis in this study confirms poorer CRF as indicated by lower VO_2peak_ in patients recovering from COVID-19 when compared to those in the non-COVID-19 group. A reduction in VO_2peak_ is possibly attributed to either one or an interplay among impaired pulmonary, cardiovascular and skeletal musculature systems (34). Given available evidence, prolonged impaired diffusing capacity of the lungs for carbon monoxide (DLCO), as a consequence of COVID-19-induced alveolar-capillary damage, plays a major role in impaired pulmonary function in discharged COVID-19 patients (35). Functional changes in the cardiac system, as reflected by a lower stroke volume (36–38), chronotropic incompetence and lower end-diastolic volume, have been observed further in COVID-19 patients (38). A recent review provided additional information that COVID-19 patients experience muscular damage, which is produced by disease-induced changes (i.e., inflammatory effects, cytokine storm and muscle catabolism), pharmacotherapy (i.e., corticotherapy) and prolonged immobility (39).

In the current meta-analysis, poorer CRF in surviving COVID-19 patients is also related to age and disease severity. Regarding age groups, our study revealed lower VO_2peak_ in all age ranges in the surviving COVID-19 group, especially in the middle-aged to older adult subgroup. Previous evidence documented that an increase in 1-metabolic equivalent (MET), as reflected by a VO_2peak_ of 3.5 mL/kg/min, associated with a 13% decrease in all-cause mortality and 15% reduction in cardiovascular disease in a healthy population (40), and 12% improvement in survival rate of the older adult (41). Based on previous evidence, this review found that a 5.31 mL reduction in VO_2peak_ in the young- to middle-aged subgroup suggests a 20% increase in all-cause mortality, 23% increase in cardiovascular disease, and 18% reduction in survival rate. These negative impacts are more pronounced in middle- to older aged COVID-19 patients, as reflected by a 15.63 mL decrease in VO_2peak_. The degree of severity in COVID-19 symptoms is also accounted for as a factor contributing to long-covid impact. This study found a lower VO_2peak_ in the COVID-19 group with moderate to severe symptoms. We note that COVID-19 survivors in advancing age had more severe symptoms whereas younger had less worsening symptoms. Lower CRF as a consequence of severe symptoms among middle- to older aged group may be explained that progressive deterioration of protective immune response is accompanied with increasing age, resulting in susceptibility to infection and experiencing more tissue-damaging inflammation (42). Obesity and physical activity status have additionally been proposed as factors for worsening symptoms (21, 43). Among included studies, COVID-19 survivors with advancing age and more severe symptoms were obese (23, 29, 30). On the other hand, younger participants with less severe symptoms had normal weight (24, 31). Our findings are in line with the previous evidence illustrating a lower CRF in hospitalized COVID-19 individuals with obesity. Excessive fat causes lower immune response via a systemic activation of hyperinflammatory promotion and the secretion of proinflammatory mediators (44). COVID-19 survivors with advancing age and worsening symptoms further had low level of physical activity. This finding is in line with a recent study that reported low perception of performing activity reflected by a poorer physical component score of health-related quality of life in severe COVID-19 patients (45). Large epidemiology has also documented that physical activity was inversely associated with severe COVID-19 (46). Physical activity is accounted as an efficient intervention for reversing an inflammatory process (47) and cardioprotective effect in COVID-19 survivors (43). In the context of current review, sufficient physical activity (≥150 min a week of moderate intensity) can impede viral entry to the targeted cell by increase in plasma soluble angiotensin-converting enzyme 2 (sACE2), which can bind to SARS-CoV-2 (43). A previous study has also supported a cardioprotective effect of a higher physical activity level as they found a significantly greater ventilatory efficient and tissue oxygen uptake in elite athletes than recreational athletes (48). Interestingly, the meta-analysis in this study failed to identify lower CRF in patients with severe symptoms when compared to the non-COVID-19 group. It is possible that those patients were in the middle-aged range, in which patients are likely to recover more quickly (23, 29). Based on the findings in this study, it was speculated that the detrimental effect of COVID-19 infection on CRF is down to coexistence of age and symptoms rather than symptoms alone.

Although the current meta-analysis showed poorer CRF among COVID-19 survivors, confidence of evidence may be reduced as it had low certainty of evidence. Low certainty suggests COVID-19 survivors may have poorer CRF than non-COVID-19 group. Decrease in the evidence certainty is attributed to serious inconsistency due to considerably heterogeneity and serious imprecision due to wide ranges of confident interval. In addition, the confidence that poorer CRF in COVID-19 survivors with getting older and more severe symptoms are likely to reduce due to very low certainty. It is uncertain whether older and severe symptoms are prone to diminish CRF in COVID-19 survivors. Factors contributing to rating down the certainty are serious inconsistency, serious imprecision, and serious risk of bias. For the serious risk of bias, body mass index distinction between groups is accounted as a potential confounding factor (23, 29) which downgrades the certainty of evidence. BMI has been identified as one of predictors of VO_2peak_ (49). Unequal BMI may distort the true effect of COVID-19 on CRF level. Thus, it should be interpreted with caution.

This is the first study to synthesize the long-term impact of COVID-19 on CRF alteration. Possible biases were eliminated by carrying out comprehensive and exhaustive searches, reviewing all available languages, and performing a review process independently by the reviewers. Furthermore, the effect estimates on CRF are likely valid, as quality of all the studies was fair to good (for case–control and cohort studies) and met the ≥70% criteria required (for cross-sectional studies). Despite the strength of this study, there were some limitations that should be considered. First, apparent heterogeneity (*I^2^* = 93.9%) across the studies may have limited reporting, as it might have had some clinical or methodological heterogeneity. This was resolved with the randoms effect model and subgroup analysis that explored the possible source of heterogeneity. Second, two of the included studies reported an estimated VO_2peak_, which might reflect an unreal VO_2peak_. Third, COVID-19 symptoms and their duration were not identified clearly in some of the studies, which may account for confounding factors influencing statistical analysis. Last, the precise effect may be different from the estimated effect due to low to very low certainty of evidence.

## 5. Conclusion

The current meta-analysis concluded that the COVID-19 group had poorer CRF than the non-COVID-19 group. Poorer CRF is likely to be more pronounced in the COVID-19 group that is getting older and having severe symptoms. The findings in this study pointed out the need to include the measurement of CRF status in clinical practice, provide suitable intervention for improving CRF and quality of life, and reduce all-cause mortality.

For a practical implication, the available evidence was limited to conclusively recommend or deny carrying out CRF assessment in COVID-19 survivors. Low certainty of evidence limits confidence about poorer CRF in COVID-19 survivors. Very low certainty of evidence also restricts the confidence of lower CRF in COVID-19 survivors who are getting older and having severe symptoms. Further well-designed research and much more data are required to prove this issue.

## Data availability statement

The original contributions presented in the study are included in the article/Supplementary material, further inquiries can be directed to the corresponding author.

## Author contributions

BC, SK, PS, and SN: conceptual framework, and drafting and revising manuscript. BC and SK: search strategy. BC, SK, and SN: study selection, risk of bias, and quality assessment. PS and SN: data extraction. BC and SN: statistical analysis. All authors contributed to the article and approved the submitted version.
